# Cinematic rendering of [^18^F]FDG-PET/MR

**DOI:** 10.1007/s00259-024-06812-9

**Published:** 2024-07-01

**Authors:** Martin W. Huellner, Klaus Engel, Grégoire B. Morand, Bernd Stadlinger

**Affiliations:** 1 Department of Nuclear Medicine, University Hospital Zurich, University of Zurich, Raemistrasse 100, Zurich, CH-8091 Switzerland; 2 Siemens Healthineers, Erlangen, Germany; 3 Department of Otolaryngology-Head and Neck Surgery, University Hospital Zurich, University of Zurich, Zurich, Switzerland; 4 Clinic of Cranio-Maxillofacial and Oral Surgery, Center for Dental Medicine, University of Zurich, Zurich, Switzerland


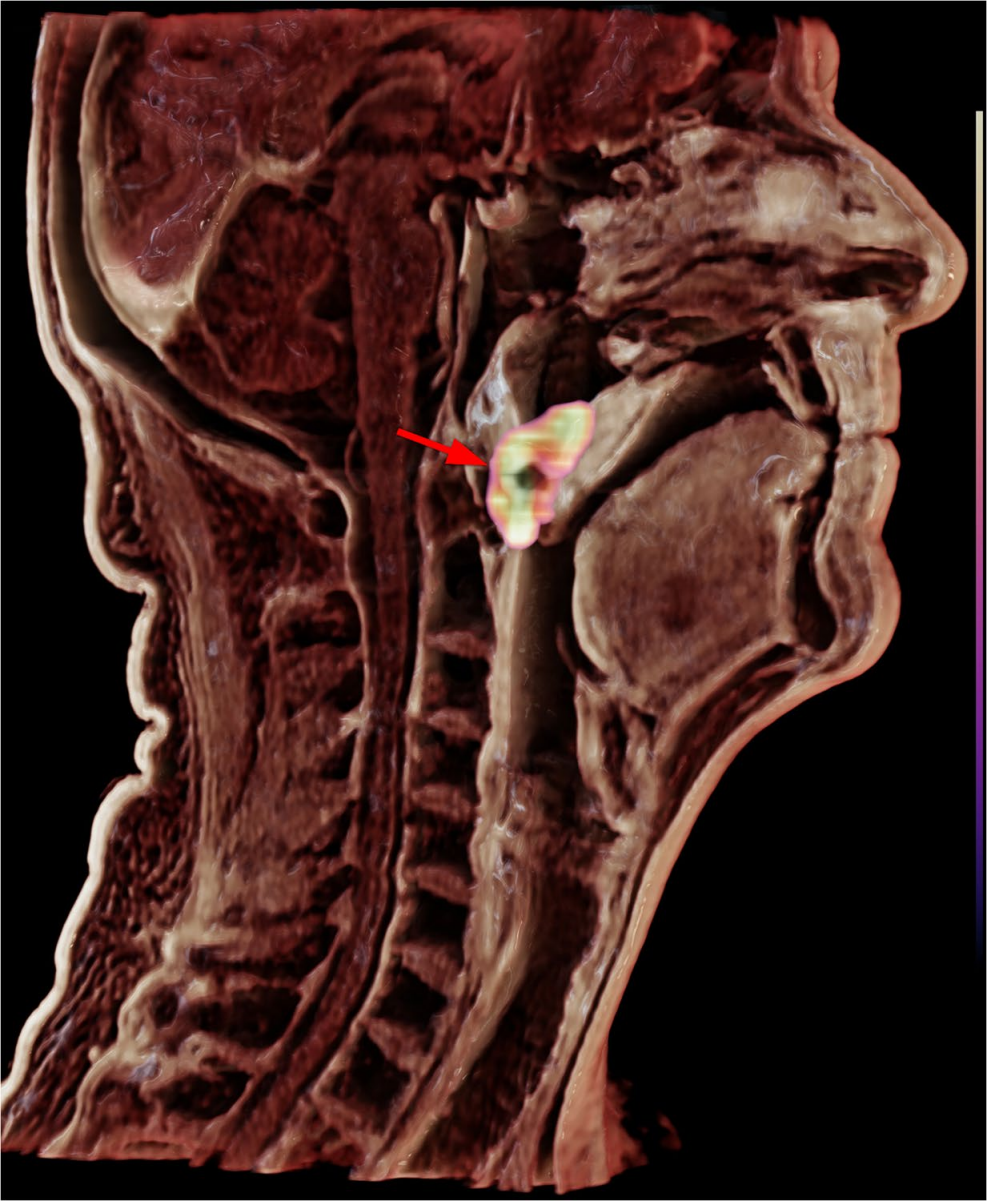


Cinematic rendering represents a transformative technique, altering the manner in which radiologists and other medical specialists perceive anatomical details and pathological conditions. Developed as a fusion of radiology and cinematography, cinematic rendering transcends traditional rendering methods, providing lifelike representations that bridge the gap between medical images and reality [1].

Cinematic rendering is a post-processing technique rooted in the segmentation of standard radiological images into photorealistic three-dimensional representations, facilitating spatial orientation [2]. Historically, its application has primarily centered on CT images [2–5], with sporadic instances noted in PET/CT [2]; however, its use in PET/MR remains unreported to date.

This image shows a cinematically rendered standard clinical [^18^F]FDG-PET/MR image (Signa PET/MR, GE HealthCare, Waukesha; MR image: T1-weighted LAVA-flex, repetition time 4.65 ms, echo time 1.82 ms, flip angle 15°, slice thickness 1.3 mm, spacing 2.6 mm, field of view 100 mm, total acquisition time 1:31 min; PET image: 245 MBq of [^18^F]FDG, bed time 1:30 min, reconstructed using block-sequential regularized expectation maximization) in a patient with an [^18^F]FDG-avid oropharyngeal carcinoma arising in the right-sided soft palate and palatine tonsil (arrow).

The cinematically rendered image presented here was retrospectively reconstructed from a standard clinical image dataset acquired within less than 2 min, using the software Cinematic Anatomy [1, 3, 6]. Such photorealistic images could potentially aid radiologists in reporting and surgeons in preoperative planning by offering crucial anatomical landmarks, enhancing visualization of subsurface structures, and complementing endoscopic images.
